# Kappa Values in Testing the Concordance: Comments on a Recent Article about Nasopharyngeal Swabs for SARS-CoV-2 Detection

**DOI:** 10.1128/spectrum.01582-22

**Published:** 2022-11-01

**Authors:** Ming Li, Chi Zhang, Tianfei Yu

**Affiliations:** a College of Computer and Control Engineering, Qiqihar University, Qiqihar, China; b Heilongjiang Provincial Key Laboratory of Resistance Gene Engineering and Protection of Biodiversity in Cold Areas, Qiqihar, China; c College of Life Science and Agriculture and Forestry, Qiqihar University, Qiqihar, China; University of Cincinnati

**Keywords:** SARS-CoV-2, COVID-19, saliva, NP swab, kappa value

## LETTER

We read with great interest the article by Callahan et al. (1) in *Microbiology Spectrum* on 18 August 2021.

In order to clarify self-collected saliva as an alternative to nasopharyngeal (NP) swabs for sample acquisition in molecular testing for severe acute respiratory syndrome coronavirus 2 (SARS-CoV-2), a total of 385 pairs of NP and saliva specimens were obtained and tested on two high-sensitivity reverse transcriptase PCR (RT-PCR) platforms, the Abbott Alinity m and Abbott m2000 (both with limits of detection [LOD] of 100 copies of viral RNA/mL). The authors found that concordance between saliva and NP swabs was excellent overall (Cohen’s *κ *= 0.93) for both initial and follow-up testing, for both platforms, and for specimens treated with guanidinium transport medium as a preservative as well as for untreated saliva (*κ *= 0.88 to 0.95). Furthermore, viral loads were on average 16× higher in NP specimens than in saliva specimens, suggesting that only a small fraction of outpatients (~8% in this study) who present with low viral loads (<1,600 copies/mL from NP swabs) would be missed by testing saliva instead of NP swabs when using sensitive testing platforms. The authors have demonstrated that the use of saliva for SARS-CoV-2 molecular detection is both feasible and practical, given suitable specimen collection containers and transport and processing protocols.

Although the authors have provided valuable information, some substantial results that may lead to misinterpretation need to be clarified. Unlike the authors, when we calculated the concordance between saliva and NP swabs by using Cohen’s kappa with the SPSS 18 statistical package (SPSS, Inc., Chicago, IL, USA) software, almost all the kappa values were different from the authors’ (Table 1). Although these questionable kappa values hardly affect the conclusions, given that accurate analysis of results is a prerequisite for scientific conclusions, we recommend the authors could explain their results in detail and clarify the misunderstanding.

**TABLE 1:** The kappa values for calculating concordance between saliva samples and NP swab samples in Callahan et al. (1) and in our analysis^*a*^

Panel in Fig. 4 from Callahan et al. (1)	Result for saliva samples	No. of NP samples with indicated result		Kappa value from:	
				Callahan et al. (1)	Our analysis
a	All (*n* = 385)	+	−		
	+	71	5	0.93	0.93
	−	4	305		
b	Initial (*n *= 343)	+	−	0.93	0.91
	+	59	5		
	−	4	275		
c	Follow up (*n *= 42)	+	−	0.88	1.00
	+	12	0		
	−	0	30		
d	Treated (*n *= 316)	+	−	0.92	0.93
	+	60	4		
	−	3	249		
e	Untreated (*n *= 69)	+	−	0.95	0.90
	+	11	1		
	−	1	56		
f	Alinity m (*n *= 207)	+	−	0.91	0.95
	+	33	2		
	−	1	171		
g	m2000 (*n *= 178)	+	−	0.94	0.91
	+	38	3		
	−	3	134		

a The data are from the article published by Callahan et al. (1).

## References

[1] Callahan C, Ditelberg S, Dutta S, Littlehale N, Cheng A, Kupczewski K, McVay D, Riedel S, Kirby JE, Arnaout R. 2021. Saliva is comparable to nasopharyngeal swabs for molecular detection of SARS-CoV-2. Microbiol Spectr 9:e00162-21. doi:10.1128/Spectrum.00162-21. PMC8552668 34406838

